# Supplementary material for: The effect of landscape on functional connectivity and shell shape in the land snail Humboldtiana durangoensis
Source: PeerJ. 2020 May 20;8:e9177. doi: 10.7717/peerj.9177 (PMC7245337; doi:10.7717/peerj.9177)
Supplement: Supplemental Information 1 — Accuracy and estimated surface covered of the Random Forest (RF) model prediction for each one of the five vegetation types considered in this study for the three different temporal frames, Last Glacial Maximum (LGM), Mid Holocene and Current. [file peerj-08-9177-s001.docx]

Table S1. Accuracy and estimated surface covered of the Random Forest (RF) model prediction for each one of the five vegetation types considered in this study for the three different temporal frames, Last Glacial Maximum (LGM), Mid Holocene and Current

| **Class** | **LGM** | | **mid Holocene** | | **Current** | | **INE INEGI (1997)** |
| --- | --- | --- | --- | --- | --- | --- | --- |
|  | **Area (km)** | **Accuracy (%)** | **Area (km)** | **Accuracy (%)** | **Area (km)** | **Accuracy (%)** | **Area (km)** |
| **Temperate forests** | 34172.003 | 59 | 39403.733 | 58 | 38279.168 | 57 | 61085.809 |
| **Cold temperate forests** | 24560.965 | 74 | 23152.255 | 73 | 24383.310 | 73 | 44189.951 |
| **Grasslands** | 38765.428 | 73 | 29008.833 | 75 | 31279.665 | 73 | 12712.325 |
| **Tropical forests** | 14787.873 | 94 | 20126.483 | 94 | 16158.300 | 95 | 6689.697 |
| **Dry zones** | 21691.513 | 86 | 22286.478 | 88 | 23877.338 | 84 | 9299.998 |
