# Supplementary figures and images for: The effect of landscape on functional connectivity and shell shape in the land snail Humboldtiana durangoensis
Source: PeerJ. 2020 May 20;8:e9177. doi: 10.7717/peerj.9177 (PMC7245337; doi:10.7717/peerj.9177)

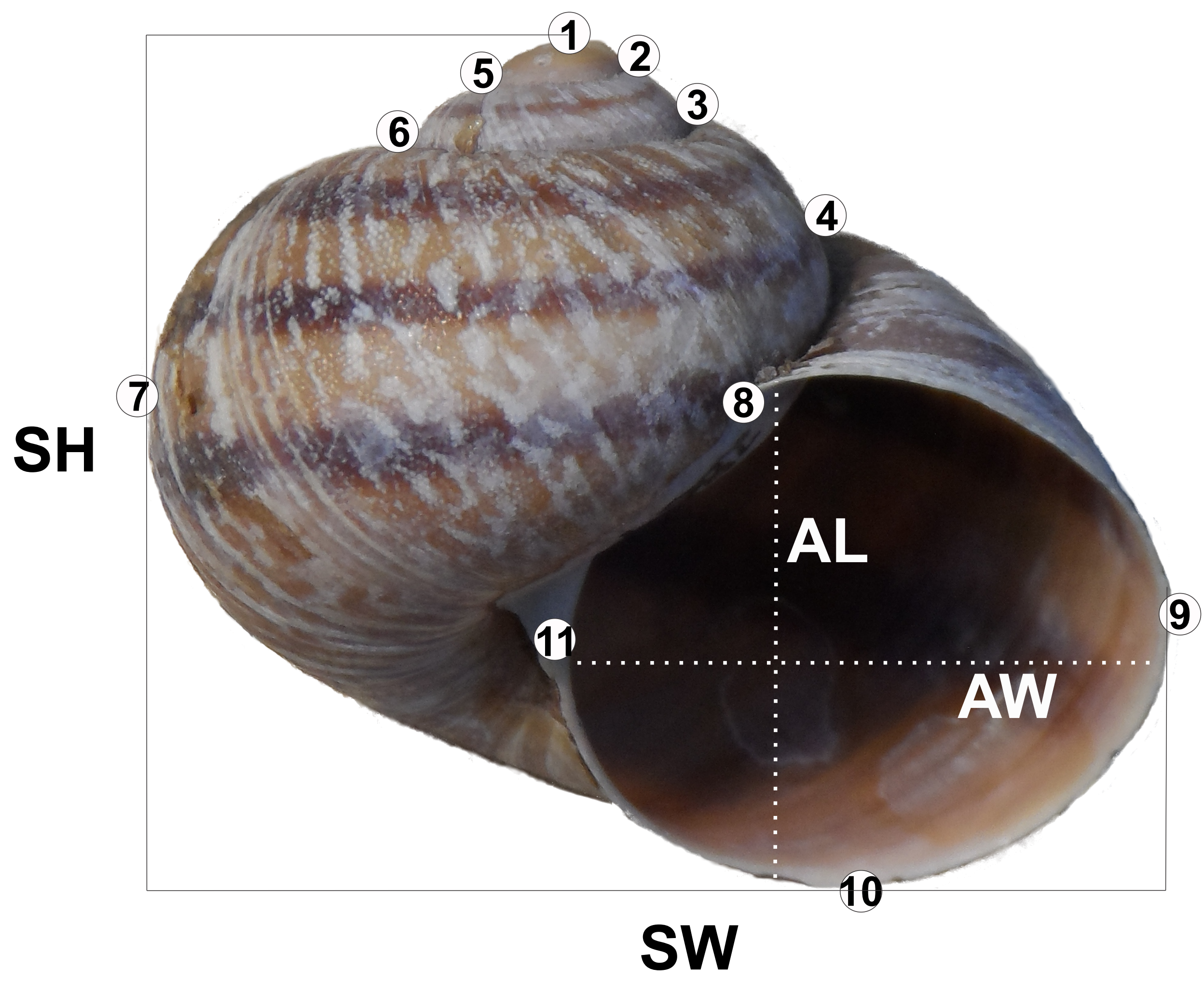

Supplement: Supplemental Information 2 [file peerj-08-9177-s002.pdf]
