# Supplementary material for: The effect of landscape on functional connectivity and shell shape in the land snail Humboldtiana durangoensis
Source: PeerJ. 2020 May 20;8:e9177. doi: 10.7717/peerj.9177 (PMC7245337; doi:10.7717/peerj.9177)
Supplement: Supplemental Information 3 [file peerj-08-9177-s003.pdf]

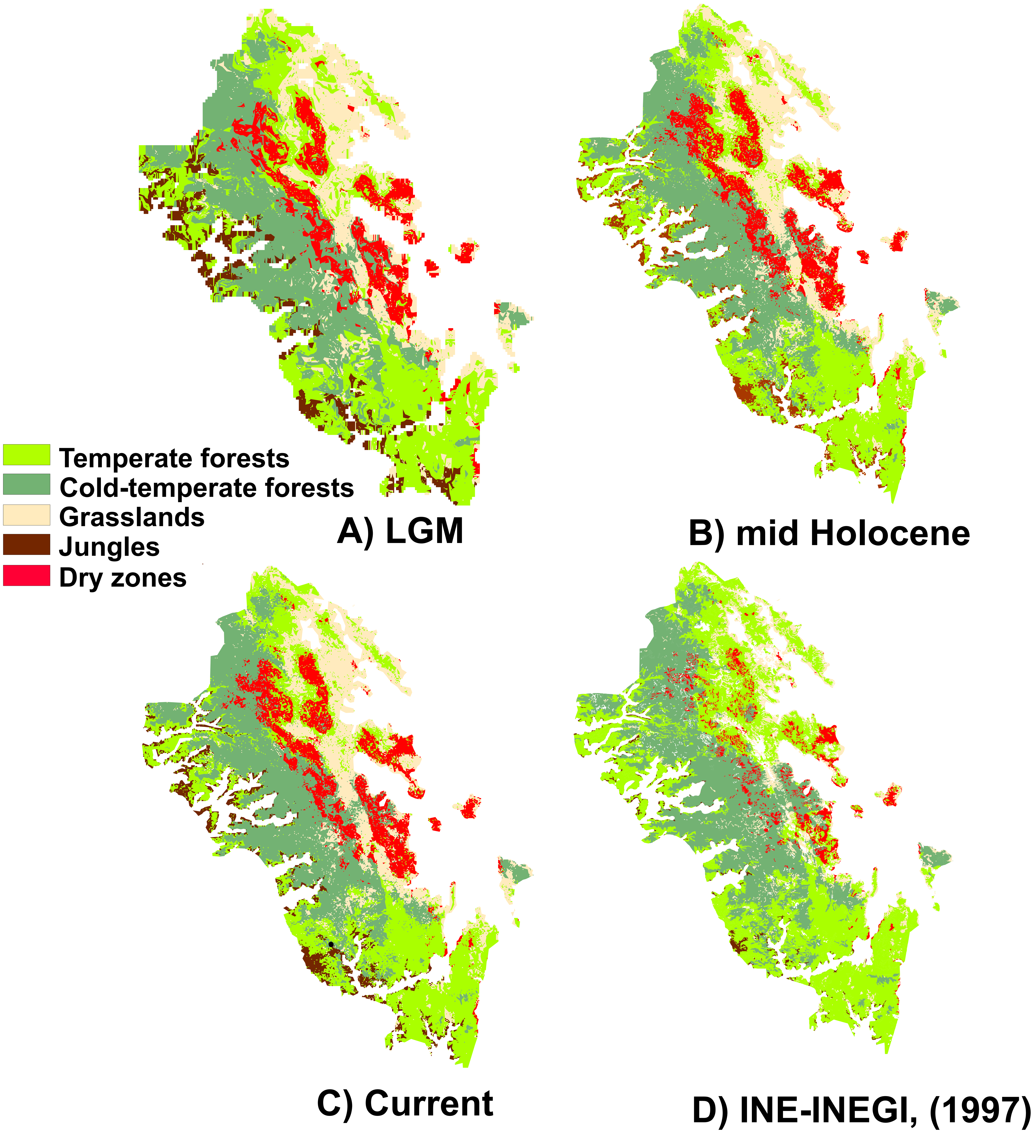
Temperate forests
Cold-temperate forests
Grasslands
Jungles
Dry zones
A) LGM
B) mid Holocene
C) Current
D) INE-INEGI, (1997)
