# Supplementary material for: The effect of landscape on functional connectivity and shell shape in the land snail Humboldtiana durangoensis
Source: PeerJ. 2020 May 20;8:e9177. doi: 10.7717/peerj.9177 (PMC7245337; doi:10.7717/peerj.9177)
Supplement: Supplemental Information 4 — The values of resistance range from low (brown) to high (blue). A to C models obtained from climate variables and D to F models obtained from vegetation cover. The circles represent the geographic centroid for each one of the seven genetic groups: 1) Guanaceví 2) Los Herreras 3) Potrero 4),Topia 5) Progreso 6) El Salto and 7) Las Peñas. [file peerj-08-9177-s004.pdf]

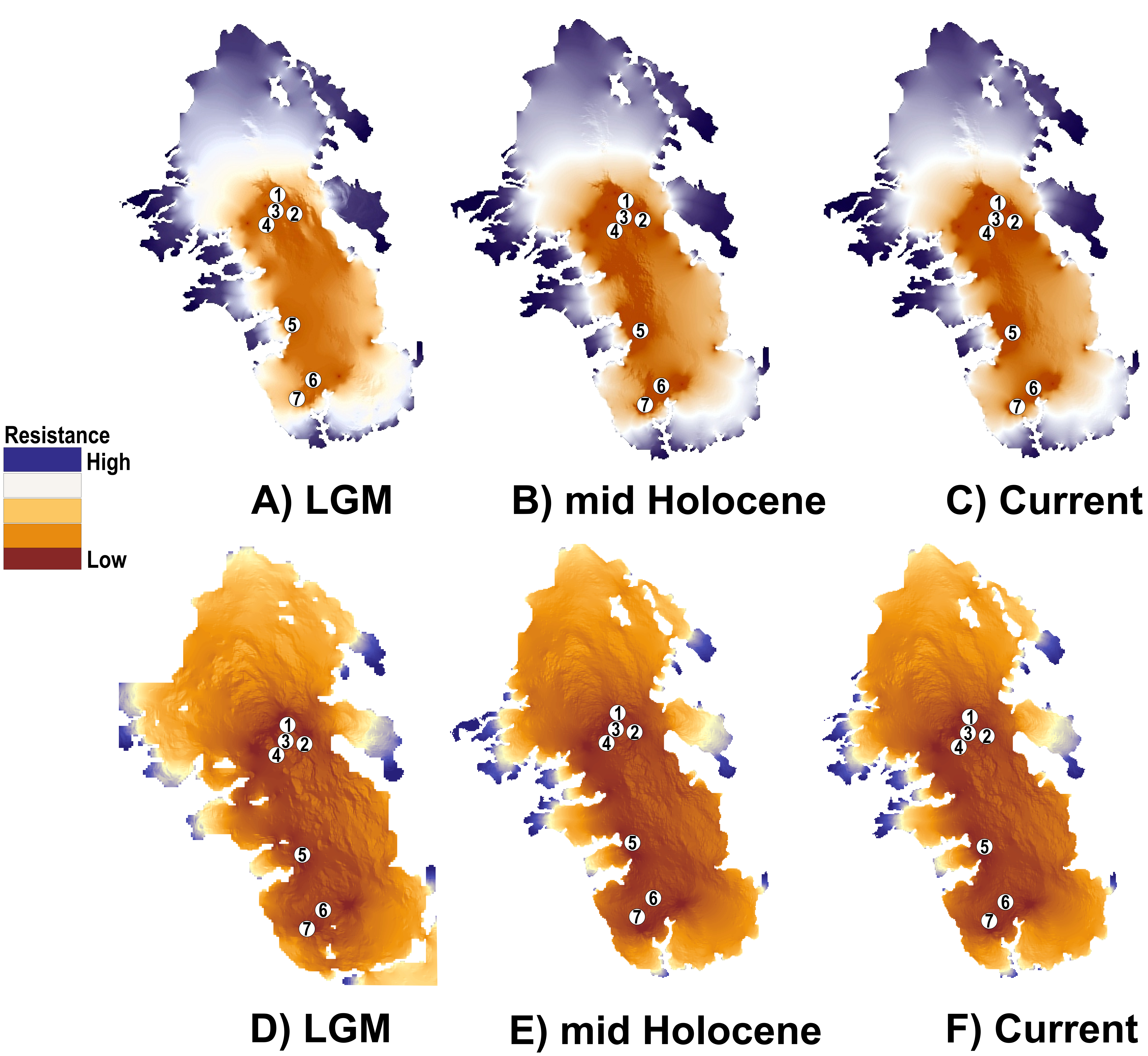
Resistance
High
Low
1
2
3
4
5
6
7
A) LGM
B) mid Holocene
C) Current
D) LGM
E) mid Holocene
F) Current
